# Electroencephalographic effects of ketamine on power, cross-frequency coupling, and connectivity in the alpha bandwidth

**DOI:** 10.3389/fnsys.2014.00114

**Published:** 2014-07-01

**Authors:** Stefanie Blain-Moraes, UnCheol Lee, SeungWoo Ku, GyuJeong Noh, George A. Mashour

**Affiliations:** ^1^Department of Anesthesiology, Center for Consciousness Science, University of Michigan Medical SchoolAnn Arbor, MI, USA; ^2^Department of Anesthesiology, Asan Medical Center, University of Ulsan College of MedicineSeoul, South Korea; ^3^Neuroscience Graduate Program, University of Michigan Medical SchoolAnn Arbor, MI, USA

**Keywords:** ketamine, consciousness, general anesthesia, anesthetic-induced unconsciousness, anesthetic mechanisms

## Abstract

Recent studies of propofol-induced unconsciousness have identified characteristic properties of electroencephalographic alpha rhythms that may be mediated by drug activity at γ-aminobutyric acid (GABA) receptors in the thalamus. However, the effect of ketamine (a primarily non-GABAergic anesthetic drug) on alpha oscillations has not been systematically evaluated. We analyzed the electroencephalogram of 28 surgical patients during consciousness and ketamine-induced unconsciousness with a focus on frontal power, frontal cross-frequency coupling, frontal-parietal functional connectivity (measured by coherence and phase lag index), and frontal-to-parietal directional connectivity (measured by directed phase lag index) in the alpha bandwidth. Unlike past studies of propofol, ketamine-induced unconsciousness was not associated with increases in the power of frontal alpha rhythms, characteristic cross-frequency coupling patterns of frontal alpha power and slow-oscillation phase, or decreases in coherence in the alpha bandwidth. Like past studies of propofol using undirected and directed phase lag index, ketamine reduced frontal-parietal (functional) and frontal-to-parietal (directional) connectivity in the alpha bandwidth. These results suggest that directional connectivity changes in the alpha bandwidth may be state-related markers of unconsciousness induced by both GABAergic and non-GABAergic anesthetics.

## Introduction

Ketamine is an anesthetic drug that was introduced into clinical practice in the 1960s (Corssen and Domino, 1966) and is currently used for the induction of unconsciousness and, at subanesthetic doses, the prevention of acute pain or the treatment of depression. Ketamine is unique in the class of general anesthetics for a number of reasons. At the molecular level, the γ-aminobutyric acid (GABA)_A_ receptor is not the primary target for ketamine, unlike many drugs used for the induction and maintenance of general anesthesia. Rather, ketamine is thought to act by antagonizing glutamatergic N-methyl-D-aspartate (NMDA) receptors (like the related anesthetics nitrous oxide and xenon) and/or hyperpolarization-activated cyclic-nucleotide gated (HCN)1 channels (Yamamura et al., 1990; Chen et al., 2009; Zhou et al., 2013). At the neurochemical level, ketamine is unique because it increases cortical acetylcholine levels and appears to depend on noradrenergic signaling for its effects, in contrast to a number of GABAergic anesthetics (Kikuchi et al., 1997; Kushikata et al., 2011). At the systems neuroscience level, ketamine is also distinct because it does not metabolically activate the ventrolateral preoptic nucleus, a sleep-promoting nucleus in the hypothalamus that is activated by commonly-used anesthetics such as propofol and isoflurane; instead, it activates the wake-promoting locus coeruleus (Lu et al., 2008). Furthermore, in contrast to virtually all other anesthetic and sedative drugs, ketamine does not appear to metabolically depress the thalamus (Långsjö et al., 2005). Finally, at the neurophysiologic level, ketamine tends to increase the power of high-frequency electroencephalographic activity, whereas most anesthetics depress this bandwidth (Maksimow et al., 2006). Ketamine therefore fails to conform to virtually all mechanistic frameworks of anesthetic-induced unconsciousness.

Despite the many unique characteristics of ketamine, we recently demonstrated that anesthetic doses of ketamine selectively inhibit frontal-to-parietal directed connectivity, as measured by symbolic transfer entropy in electroencephalographic signals (Lee et al., 2013b). This finding was notable because selective inhibition of effective and directional connectivity from frontal to parietal regions has been demonstrated for two commonly-used anesthetics that act (at least in part) through GABA receptors: propofol [demonstrated with symbolic transfer entropy, evolution map approach, dynamic causal modeling, directed phase lag index (dPLI)] and sevoflurane (demonstrated with symbolic transfer entropy, evolution map approach) (Lee et al., 2009, 2013a; Ku et al., 2011; Boly et al., 2012). Given the proposed role of top-down reentrant processing and frontal-parietal networks in the conscious perception of environmental stimuli (Dehaene and Changeux, 2011; Demertzi et al., 2013), this finding suggests the exciting possibility of a common neurobiology underlying anesthetic-induced unconsciousness.

In the current study, we further characterized the effects of ketamine on the electroencephalogram, with a focus on alpha rhythms (8–14 Hz). Alpha oscillations have been an active area of research into anesthetic-induced unconsciousness because of a characteristic process referred to as *anteriorization*. In the resting/eyes-closed state in humans, the electroencephalographic power of alpha is dominant over the occipital cortex. However, at the point of propofol-induced unconsciousness, the power of alpha is dominant over the frontal cortex (Feshchenko et al., 2004; Purdon et al., 2013). Computational models that account for the observed posterior-to-anterior shift of alpha rhythms are based on propofol's agonism of GABA_A_ receptors in the thalamus (Ching et al., 2010; Vijayan et al., 2013; Ching and Brown, 2014). The anteriorization of alpha at the point of propofol-induced unconsciousness also appears to relate to a characteristic cross-frequency coupling pattern between the amplitude of alpha and the phase of slow oscillations. During transitions into and out of propofol-induced unconsciousness, there is a trough-max relationship, i.e., the maximal alpha amplitude is coupled to the trough of the slow oscillation. During deeper levels of propofol-induced unconsciousness, however, this coupling shifts to a peak-max relationship (Purdon et al., 2013; Mukamel et al., 2014). These coupling patterns have not been investigated during unconsciousness induced by ketamine or other non-GABAergic anesthetics.

The propofol-induced shift to hypersynchronous alpha in the frontal cortex has been posited to impair flexible corticocortical communication (Supp et al., 2011), a process thought to be critical for consciousness. This hypothesis is supported by our recent work demonstrating that propofol-induced unconsciousness is characterized by a depression of anterior-to-posterior corticocortical connectivity (as measured by dPLI) that is found only in the alpha bandwidth (Lee et al., 2013a). dPLI is a measure of directional connectivity that appears to be closely related to alpha, since neural mass models of the human brain demonstrate an anterior-to-posterior flow of dPLI in the alpha bandwidth (Stam and van Straaten, 2012). This finding has been confirmed empirically by our previous study of dPLI in the resting state of conscious human volunteers (Lee et al., 2013a).

The potential dependence of anteriorized alpha rhythms on GABA_A_ effects in the thalamus leads to the prediction that a non-GABAergic drug like ketamine would differ significantly from propofol and not increase the power of frontal alpha rhythms in association with the induction of unconsciousness. Similarly, we would not predict any characteristic cross-frequency coupling patterns around the time of unconsciousness, since these relationships appear to depend on increases in the frontal power of alpha. However, if the unconscious states induced by ketamine and propofol nonetheless share an underlying neurobiology, we would predict that the directional connectivity measure of dPLI would be inhibited by ketamine (as is observed with propofol), both in terms of the specificity of the alpha bandwidth and the anterior-to-posterior directionality. We tested these predictions regarding the effects of ketamine on alpha oscillations by re-analyzing electroencephalographic data acquired during consciousness and ketamine-induced unconsciousness in human surgical patients.

## Methods

### Participants

We collected electroencephalographic data from 30 patients undergoing elective stomach, colorectal, thyroid or breast surgery (15 males; American Society Anesthesiologists Physical Status 1 or 2; 22–64 years old) at the Asan Medical Center (Seoul, South Korea). This study was approved by the Institutional Review Board of Asan Medical Center; written consent was provided by all participants after a careful discussion of risks and benefits. Patients were excluded from the study if they had a history of cardiovascular disease (including hypertension), brain surgery, drug/alcohol dependence, neurological or psychiatric disorder, or if they were currently using psychotropic medication. The data collected were previously analyzed and published (Lee et al., 2013b); the current study uses distinct analytic methods to test distinct hypotheses. For this secondary analysis, two participants were excluded due to insufficient artifact-free data in the baseline recording period; accordingly, data from 28 participants were analyzed at the University of Michigan Medical School (Ann Arbor, MI) for the current study.

### Anesthetic protocol

Unconsciousness was induced using an infusion of ketamine (2 mg/kg diluted in 10 mL of 0.9% normal saline) over a 2 min period (Baxter infusion pumpAS40A, Baxter Healthcare Corporation, Deerfield, IL). Consciousness was monitored by assessing the participant's response to an auditory command (“squeeze your right hand twice”), which was repeated every 10 s. Prior to loss of consciousness, no sedatives or other medications were administered to the participant. Standard intraoperative monitors (non-invasive blood pressure measurement, electrocardiography, pulse oximetry and end-tidal carbon dioxide concentration and non-invasive) were used throughout the experiment; if systolic blood pressure increased to over 30% of baseline values, 5–10 mg of labetalol were administered.

### Electroencephalogram data acquisition

The electroencephalogram was acquired at 8 electrodes located at Fp1, Fp2, F3, F4, T3, T4, P3, and P4 (International 10–20 system); all channels were referenced to A2. Electrode impedances were reduced to below 5 KΩ prior to data collection and electroencephalographic signals were collected used a Laxtha WEEG-32 amplifier (LXE3232-RF, Laxtha Inc., Daejeon, Korea) with a sampling frequency of 256 Hz. Data recording began 5 min prior to induction; during this period, participants were instructed to rest with their eyes closed. Data collection continued through anesthetic induction and was terminated five minutes following loss of consciousness.

### Electroencephalogram analysis

**Preprocessing:** Electroencephalogram data were bandpass filtered between 0.1 and 50 Hz. The filtered data were visually inspected and non-physiological artifacts were removed. For the spectral and cross-frequency coupling analysis, two 5-min epochs of data were extracted from every participant: (1) Baseline rest, prior to ketamine induction, and (2) Induction and unconsciousness, beginning at the start of ketamine infusion. This second epoch was chosen specifically so that the transition from consciousness to unconsciousness could be studied, as some spectrographic and phase-amplitude coupling patterns appear during this period (Purdon et al., 2013). On average, participants lost consciousness 89 ± 14 s after the start of ketamine infusion; thus, this epoch contains electroencephalographic data from both conscious and unconscious states. However, coherence, phase lag index (PLI) and dPLI measures were calculated only with the data after ketamine-induced consciousness. **Spectral Analysis:** Spectrograms were computed in Chronux (www.chronux.org), using the multitaper method, with window lengths of *T* = 2 s, step size of 0.1 s, time-bandwidth product *NW* = 2, number of tapers *K* = 3. Group spectrograms were calculated by aggregating data from frontal channels F3 and F4 across all participants. **Phase-Amplitude Coupling Analysis:** Phase-amplitude coupling was conducted using the Phase-Amplitude Coupling Toolbox (PACT) in EEGlab (Delorme and Makeig, 2004; Miyakoshi et al., 2013). Finite impulse response filters were used to extract low-frequency (0.1–1 Hz) and alpha (8–14 Hz) oscillations from channels F3 and F4 for each participant. Instantaneous phase and amplitude were extracted using a Hilbert transform. Phase-amplitude modulograms were calculated by assigning each temporal sample to one of *N* = 18 equally spaced phase bins based on the instantaneous value of the low-frequency phase and then averaging the corresponding instantaneous amplitude of alpha within a 1 min epoch. Additionally, Canolty's phase-amplitude coupling modulation index was calculated for each minute of data and averaged across all participants (Canolty et al., 2006). **Functional Connectivity Analysis with Coherence:** The magnitude squared coherence estimate was calculated between each set of channel combinations using Welch's averaged modified periodogram method (Welch, 1967). **Functional Connectivity Analysis with Phase Lag Index (PLI):** To mitigate the effects of choice of reference and volume conduction, we calculated functional connectivity using the metric PLI (Stam et al., 2007). We used a Hilbert transform to extract the instantaneous phase of the electroencephalogram from each channel and calculated the phase difference Δϕ_*t*_ between channels, where Δϕ_*t*_ = ϕ_*i,t*_ − ϕ_*j,t*_, *t* = 1, 2, …, n n is the number of samples within one epoch, and *i* and *j* were set to include all possible channel combinations. PLI was then calculated using Equation (1):

(1) $$\begin{array}{cc} {\text{PLI}}_{ij}=\left|\langle \text{sign}\left(\Delta \phi_{t}\right)\rangle \right|,\;0\leq {\text{PLI}}_{ij}\leq 1. & \left(1\right) \end{array}$$

Here, the sign() function yields: 1 if Δϕ_*t*_ > 0; 0 if Δϕ_*t*_ = 0; and −1 if Δϕ_*t*_ < 0. If the instantaneous phase of one signal is consistently ahead of the other signal, the phases are considered locked, and PLI≈ 1. However, if the signals randomly alternate between a phase lead and a phase lag relationship, there is no phase locking and PLI ≈ 0. **Directed Connectivity Analysis with Directed Phase Lag Index (dPLI):** To determine the direction of the phase-lead/phase-lag relationship between channels, we calculated dPLI between signals *i* and *j* using Equation (2) (Stam and van Straaten, 2012):

(2) $$\begin{array}{cc} {\text{dPLI}}_{ij}=\langle H(\Delta \pi_{t})\rangle & \left(2\right) \end{array}$$

Here, *H* (x) represents the Heaviside step function, where *H* (x) = 1 if *x* > 0, *H* (x) = 0.5 if *x* = 0, *and H* (x) = 0 otherwise. If, on average, signal *i* leads signal *j*, 0.5 < dPLI_*ij*_ = 1; if signal *j* leads signal *i*, 0 = dPLI_*ij*_ < 0.5; and if there is no phase-lead/phase-lag relationship between signals, dPLI = 0.5. **Surrogate Data Analysis:** To quantify the effects of spurious phase relationships and power spectrum changes on functional and directed connectivity metrics, we generated surrogate data sets as follows. We calculated the instantaneous phase of each combination of channel pairs *i* and *j* for each epoch (baseline; ketamine-induced unconsciousness) using a Hilbert transform. The phase time series of channel *i* was maintained, whereas in channel *j*, the phase time series from 0 to n/2 was interchanged with the phase time series from n/2 to n, where n is the number of samples within one epoch. In this manner, existing phase relationships were eliminated while maintaining the spectral properties of each condition. PLI and dPLI were calculated for all channel combinations in the surrogate dataset. **Brain Network Visualization:** The brain networks were visualized with the BrainNet Viewer (Xia et al., 2013) (http://www.nitrc.org/projects/bnv/). Montreal Neurological Institute (MNI) coordinates of each scalp electrode were used to generate nodes that were projected onto an axial view of the brain. Coherence, PLI, and dPLI measures were indicated by varying the color and size of edges between these nodes.

### Statistical analysis

**Phase Amplitude Coupling:** To test the null hypothesis that the phase and amplitude of the frontal electroencephalogram are decoupled, we employed the surrogate data method proposed in Canolty et al. (2006). Data were divided into 1-min epochs; within each epoch, a surrogate time series was generated by shifting the amplitude by a random time Δt (0 ≤ Δt ≤ 60 s) while keeping the phase series fixed. The modulation index was calculated for the surrogate series. This procedure was repeated 2000 times to produce distributions of modulation index values in which the null hypothesis was true. The modulation index for the experimental data was considered significant if it exceeded 95% of the surrogate values (*p* < 0.05). **Coherence, PLI, and dPLI:** Coherence, PLI, and dPLI values were compared between (1) baseline and unconscious epochs, (2) baseline and surrogate baseline epochs, and (3) unconscious and surrogate unconscious epochs using a student's *t*-test. Differences were considered significant at α < 0.05.

## Results

### Ketamine-induced unconsciousness is not characterized by increases in frontal alpha power or by phase-amplitude coupling patterns

**Figure 1:**
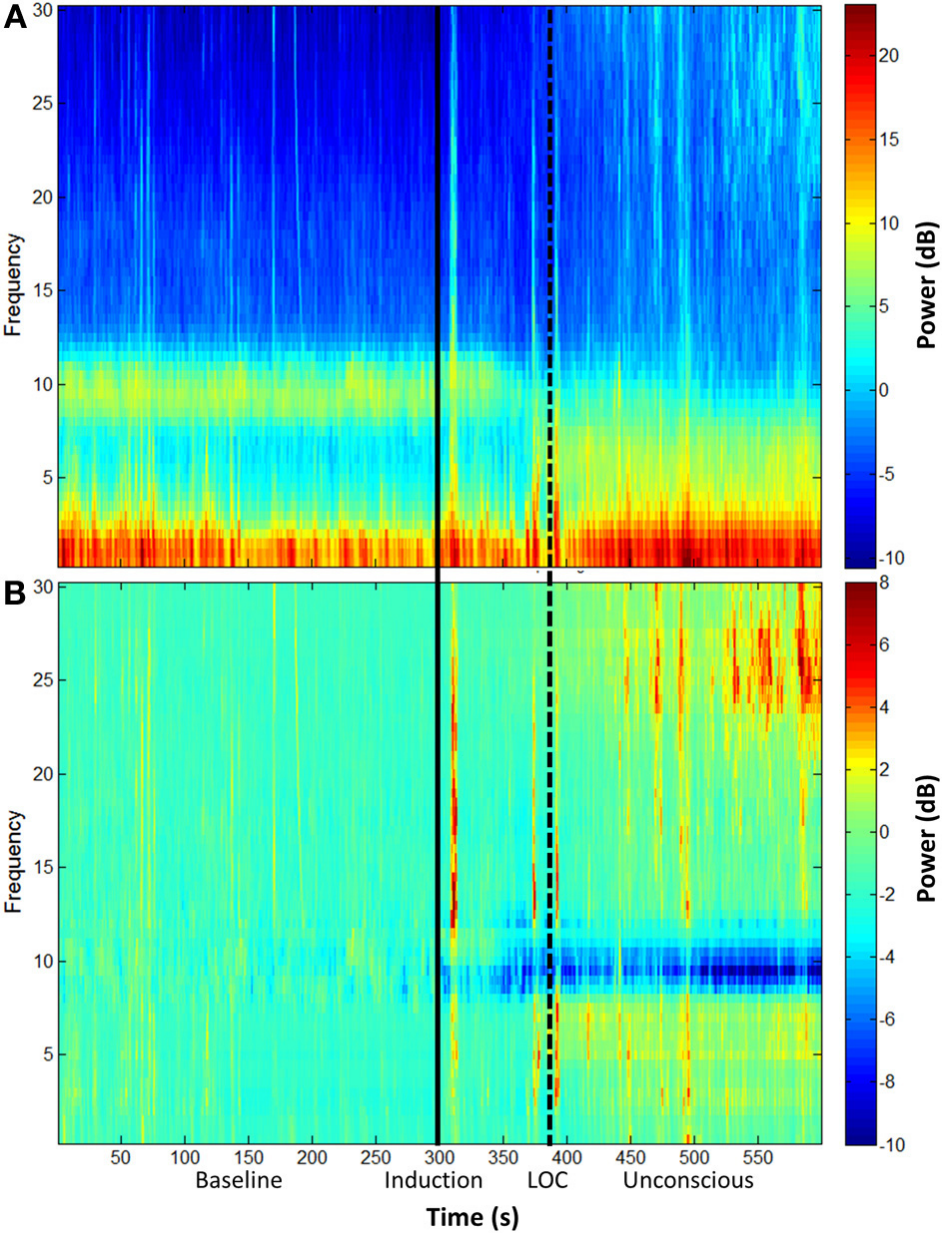
**Group spectral analysis. (A)** Spectrogram and **(B)** baseline-normalized spectrogram of frontal channels (F3 and F4) demonstrate a decrease in alpha power during and after ketamine-induced unconsciousness. LOC, loss of consciousness.

The group-level spectrogram of the electroencephalogram recorded from frontal channels demonstrates a decrease in alpha power upon ketamine-induced unconsciousness (Figure 1). This is the opposite pattern to that observed in propofol-induced unconsciousness, which is associated with an increase in alpha power (Purdon et al., 2013).

**Figure 2:**
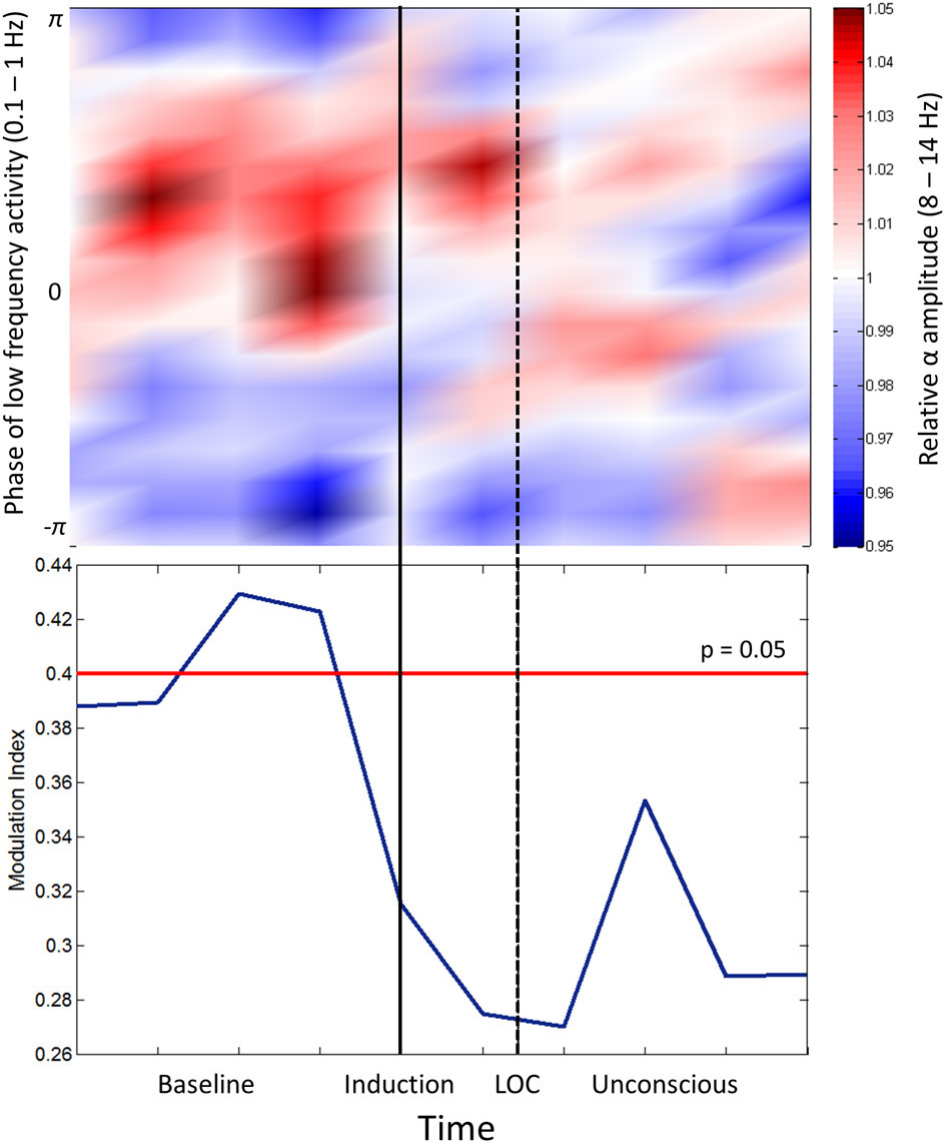
**Phase-amplitude coupling analysis. Top panel:** Frontal phase-amplitude coupling across baseline and induction/unconscious epochs. **Bottom panel**: Modulation indices (blue line) of each minute of data in baseline and induction/unconscious epochs; values are significant at *p* = 0.05 above the red line. LOC, loss of consciousness.

Cross-frequency coupling patterns were calculated between slow oscillation phase and alpha amplitude in the frontal channels. Group modulograms and the group-averaged modulation index demonstrate that a significant trough-max coupling pattern existed for 40% of the baseline resting state, but no significant coupling was observed upon induction or after loss of consciousness (Figure 2). These patterns are in contrast to those observed with propofol, where loss of consciousness is associated with a trough-max coupling pattern (Mukamel et al., 2014). It is worthwhile noting that when the test for phase-amplitude coupling significance (Canolty et al., 2006) is applied to datasets with minimal to no cross-frequency coupling patterns, epochs with even moderate coupling patterns will exceed the modulation index associated with significance. This may explain the significant phase-amplitude coupling observed during the resting state of our study, which has not been observed in previous studies.

### Ketamine-induced unconsciousness is associated with decreases in PLI, but not in coherence

**Figure 3:**
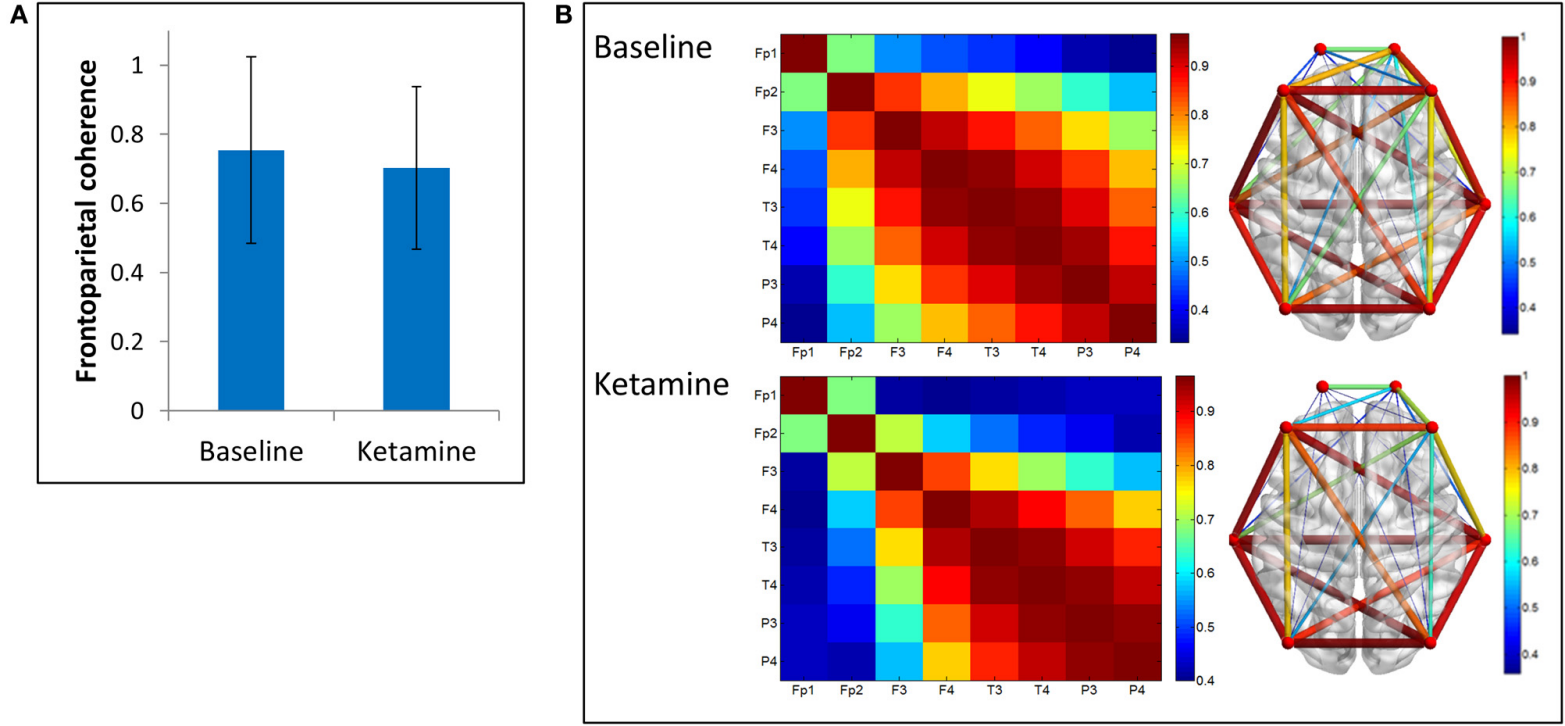
**Coherence analysis**. **(A)** Average frontoparietal coherence across 28 participants in baseline resting and ketamine-induced unconscious states (error bars indicate standard deviation). No significant differences are observed in coherence between conditions. **(B)** Average coherence across all channel combinations in baseline rest and ketamine-induced unconscious states, represented on a channel grid (left) and on an axial view of the brain (right). On the brain network, both color and size of the edges represent strength of coherence.

We examined two measures of functional connectivity in the alpha bandwidth between frontal and parietal channels: coherence and PLI. We observed no significant change in frontoparietal coherence (*p* = 0.29) between baseline resting and ketamine-induced unconsciousness (Figure 3).

**Figure 4:**
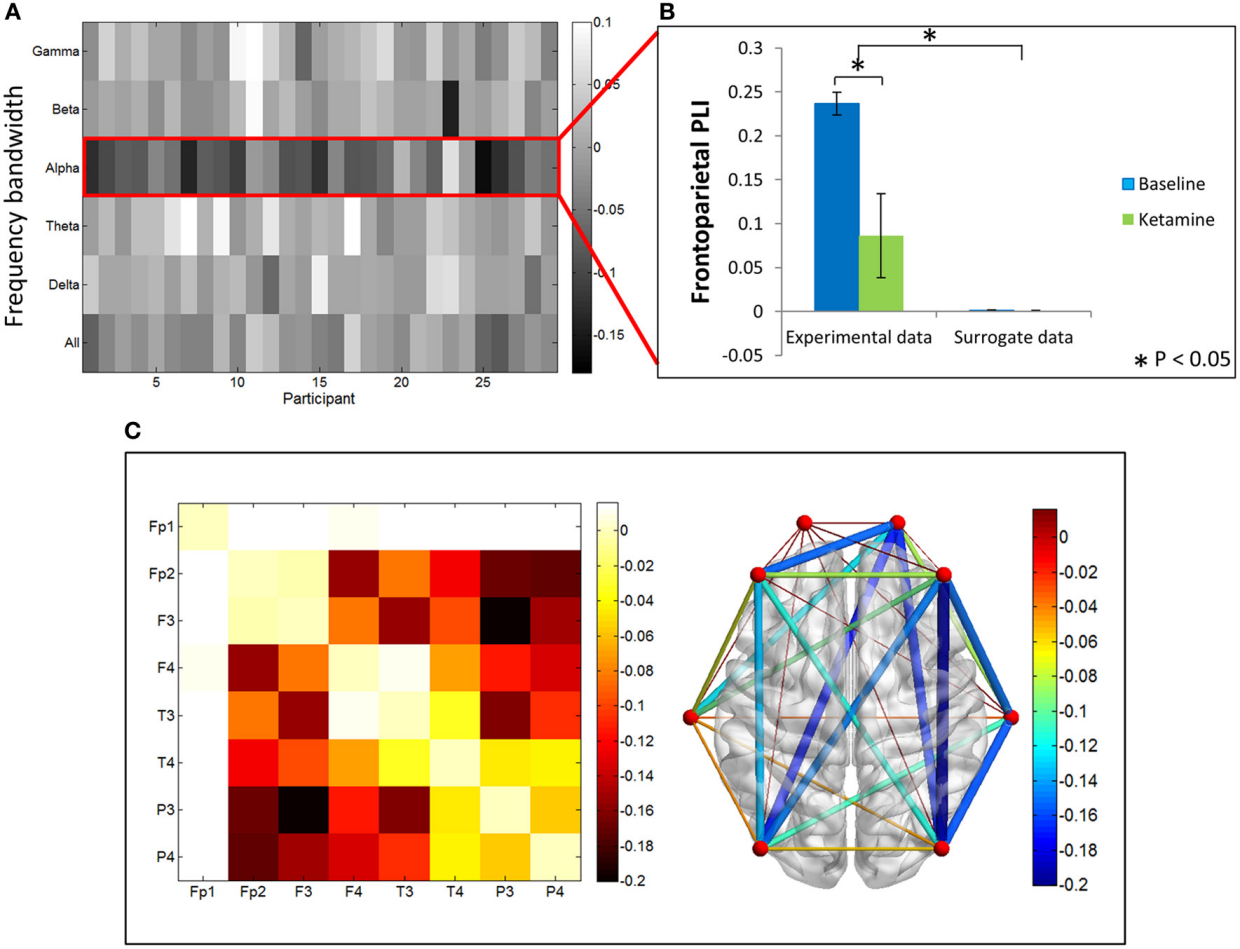
**Phase lag index analysis**. **(A)** Difference in PLI between baseline and ketamine-induced unconscious states represented for all bandwidths, across all participants. The largest decreases in PLI are observed in the alpha bandwidth. **(B)** Average frontoparietal PLI values for experimental data and surrogate data (error bars indicate standard deviation). Frontoparietal PLI significantly decreases (as indicated by ^*^) between states in the experimental data; no phase-locking is observed in the surrogate dataset. **(C)** Average difference in PLI between baseline and unconscious states across all channel combinations, represented on a channel grid (left), and on the axial view of the brain (right). Color and size of edges in the brain network represent the difference in PLI between baseline and unconscious states. Large decreases in PLI are observed between frontoparietal channel combinations.

In contrast, PLI decreased upon ketamine-induced unconsciousness. The largest decrease in PLI was observed in the alpha bandwidth, where PLI values during unconsciousness were significantly lower than PLI values calculated during baseline consciousness (*p* < 0.001) (Figures 4A,B). PLI values measured from surrogate data in both epochs were ~0, indicating that the values calculated from experimental data are not the result of spurious phase relationships. While there was an overall decrease in PLI across all channel combinations, the largest decrease occurred across frontoparietal channel pairs (Figure 4C).

### Ketamine-induced unconsciousness is associated with decreased frontoparietal dPLI

**Figure 5:**
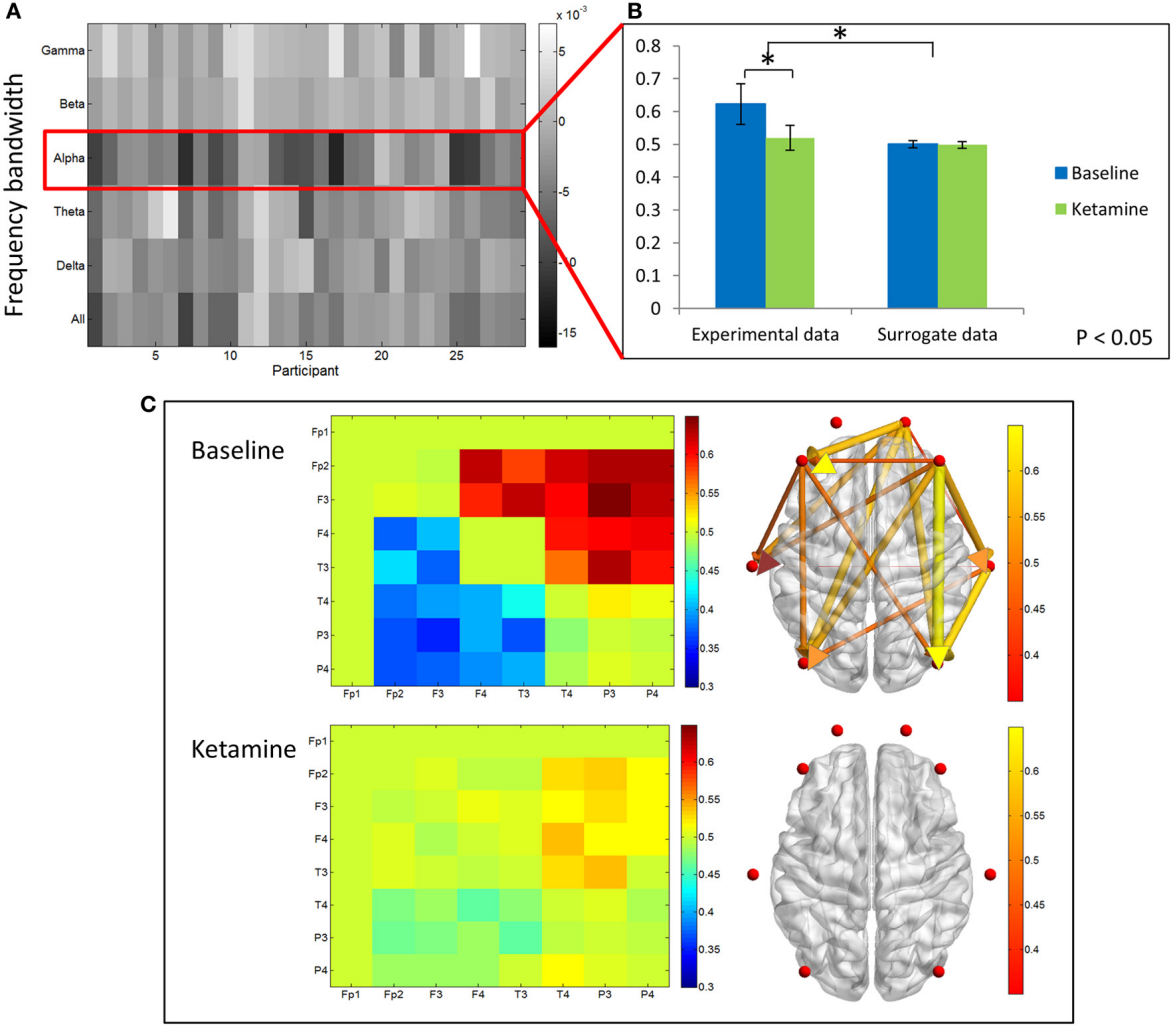
**Directed phase lag index analysis**. **(A)** Difference in frontoparietal dPLI between baseline rest and ketamine-induced unconscious states, represented across six frequency bandwidths and all participants. The largest decreases in dPLI occur in the alpha bandwidth. Note that a dPLI value of 0.5 indicates neither phase lag nor phase lead relationship. **(B)** Average frontoparietal dPLI values for each state in experimental and surrogate data (error bars indicate standard deviation). Frontoparietal dPLI significantly decreases during ketamine-induced unconsciousness (as indicated by ^*^). Surrogate data demonstrate no phase-lead/phase-lag relationship in either state. **(C)** Average dPLI values across all channel combinations in baseline and unconscious states. Only dPLI values greater than 0.55 are represented on the axial brain network. In this network, color, and size of the edges indicate the dPLI value between two nodes; arrows indicate the direction of phase lead and lag.

We examined differences in dPLI between baseline resting and unconscious states. The greatest differences in dPLI occurred in the alpha bandwidth (Figure 5A) and a significant decrease in dPLI from frontal to parietal brain regions (*p* < 0.001) was observed between states (Figure 5B). There was no significant difference between dPLI values of the surrogate data generated from either state, indicating that the observed changes in dPLI for the experimental data cannot be attributed to changes in spectral power between conditions. Baseline dPLI indicated that the dominant direction of connectivity is from frontal to parietal regions when participants are conscious; upon ketamine-induced unconsciousness there is a balance of frontoparietal and parietofrontal directional connectivity (Figure 5C).

## Discussion

This study of ketamine and alpha oscillations revealed both drug-related and state-related effects in association with the induction of unconsciousness. Unlike propofol, ketamine does not increase frontal alpha power or induce characteristic cross-frequency coupling patterns between the power of alpha and the phase of slow-wave oscillations. Functional connectivity in the alpha bandwidth was preserved between states when measured by coherence, but decreased upon unconsciousness when measured by PLI. The discrepancy between these two functional connectivity metrics indicates that while the correlation between electroencephalogram signals in frontal and parietal regions remains unchanged in aggregate, their specific phase-relationship is disrupted upon loss of consciousness. Directional connectivity in the alpha bandwidth (as measured by dPLI) was inhibited across the frontoparietal network by ketamine, a finding consistent with our past study of propofol-induced unconsciousness (Lee et al., 2013a). The identification of shared neural features between the unconscious states induced by GABAergic and non-GABAergic anesthetics has been a longstanding problem in the study of both anesthetic mechanisms and anesthetic monitoring. The current findings support the hypothesis that anesthetic-induced unconsciousness has a common neurobiology related to disrupted functional relationships across cortical or thalamocortical networks.

Activity and connectivity of lateral frontal and posterior parietal cortices have been hypothesized to be critical for consciousness of environmental stimuli (Boly et al., 2007; Demertzi et al., 2013). As such, the finding that a variety of general anesthetic drugs suppresses both activity and connectivity in these regions appears relevant to the proximate cause of anesthetic-induced unconsciousness. Prior preclinical studies of anterior-posterior connectivity in rat brain identified a selective inhibition of frontal-to-posterior transfer entropy in the gamma bandwidth in association with isoflurane-induced unconsciousness (Imas et al., 2005). Our laboratory first demonstrated anesthetic inhibition of frontal-to-parietal connectivity in human volunteers (Lee et al., 2009) and surgical patients (Ku et al., 2011); inhibition of functional, directional, and effective connectivity in frontal-parietal networks in association with propofol-induced unconsciousness has been identified by studies from multiple research groups using multiple analytic methods (Boveroux et al., 2010; Schrouff et al., 2011; Boly et al., 2012; Jordan et al., 2013). Of note, the recent study of Jordan et al used combined electroencephalography and functional magnetic resonance imaging with no *a priori* assumptions regarding connectivity and found that the selective loss of frontal-to-parietal connectivity (as measured by symbolic transfer entropy) was the best discriminator between consciousness and propofol-induced unconsciousness (Jordan et al., 2013). However, most studies of directional/effective connectivity in frontal-parietal networks have focused on propofol, a prototypical GABA_A_ agonist. Recently, our laboratory demonstrated a similar and selective inhibition of frontal-to-parietal connectivity during unconsciousness induced by the diverse anesthetics ketamine, propofol, and sevoflurane (Lee et al., 2013b). This study was the first to identify a common network-level disruption induced by both GABAergic and non-GABAergic anesthetics. The current study builds on this work by using alternative connectivity measures to support the hypothesis that anesthetic-induced unconsciousness is characterized by interrupted directed connectivity from frontal to parietal regions. Impaired connectivity is of relevance to the mechanism of anesthetic-induced unconsciousness because it reduces the likelihood of achieving the neural information synthesis thought to be necessary for conscious perception (Tononi, 2011). Furthermore, by comparing ketamine's effects on the alpha bandwidth to past studies of propofol, we are now able to distinguish between drug-related and state-related effects on the electroencephalogram. Despite the divergent spectral effects on alpha oscillations induced by ketamine in this study and those reported in past studies of propofol, disruption of functional (PLI) and directional (dPLI) connectivity in the alpha bandwidth appears common to the state of unconsciousness induced by both drugs. It should be noted that changes in dPLI were reversed upon recovery from propofol; given the current study design we were not able to assess recovery of directional connectivity for ketamine.

This study has numerous limitations. First, we reanalyzed electroencephalographic data from a prior study of ketamine-induced unconsciousness (Lee et al., 2013b). It might be argued that the current findings could have been predicted from our past methodology using symbolic transfer entropy. However, this is not necessarily the case, since analysis of the same electroencephalographic dataset (Murphy et al., 2011) with dynamic causal modeling (Boly et al., 2012) and Granger causality (Barrett et al., 2012) yielded discrepant results regarding directionality across frontal and parietal cortices. Second, we used low-resolution electroencephalography given the clinical nature of the original study; thus, source-localized signals could not be analyzed. Third, there was no recording from occipital electrodes, which precluded topographic analysis that would allow comparison of alpha power in posterior and anterior structures. Fourth, it could be argued that the state of ketamine-induced unconsciousness is substantially distinct from the state of propofol-induced unconsciousness, which renders any comparisons questionable. Although it is true that ketamine-based anesthetics can be associated with conscious states such as dreaming or hallucinations (Grace, 2003), the same could be argued—albeit to a lesser degree—for propofol (Leslie et al., 2007). Furthermore, from the functional perspective, both ketamine and propofol can be used to induce unconsciousness in clinical settings; this functional similarity motivates the search for common mechanisms. Fifth, it one could argue that the reduction in PLI and dPLI we observed in the alpha bandwidth during induction/unconsciousness can be attributed to the dramatic decrease in power upon loss of consciousness. However, we have demonstrated that PLI and dPLI values calculated for surrogate datasets that have the same spectral characteristics are not significantly different. Furthermore, the fact that coherence values are not significantly different between states demonstrates that the change in PLI and dPLI values cannot be attributed to reduced power in the alpha bandwidth. Finally, we used loss of responsiveness as a surrogate for loss of consciousness; it is well known that consciousness and responsiveness are dissociable. However, this is a limitation common to all studies of anesthetic-induced unconsciousness. Although, as noted, loss of responsiveness induced by ketamine may still be associated with conscious states such as dreams, it is the connection to the environment that is likely of primary clinical relevance (Sanders et al., 2012).

Despite these limitations, this analysis of ketamine's effects on spectral changes, cross-frequency coupling, and connectivity in the alpha bandwidth provides insight into the neurobiology of ketamine-induced unconsciousness. In comparison with past studies of propofol, the distinct effects of ketamine suggest that the ability to induce anteriorized alpha and cross-frequency coupling varies across anesthetic drugs. By contrast, disrupted directional connectivity in the frontal-parietal network may be a common state-related feature—and, potentially, a common mechanism—of anesthetic-induced unconsciousness.

## Funding

Supported by departmental and institutional funds.

### Conflict of interest statement

Dr. George A. Mashour and Dr. UnCheol Lee have a patent pending (through the University of Michigan, Ann Arbor) on the measurement of directional connectivity for monitoring consciousness. The authors declare that the research was conducted in the absence of any commercial or financial relationships that could be construed as a potential conflict of interest.
